# The Influence of the Binder Phase on the Properties of High-Pressure Sintered Diamond Polycrystals or Composites for Cutting Tool Applications

**DOI:** 10.3390/ma18030634

**Published:** 2025-01-30

**Authors:** Lucyna Jaworska

**Affiliations:** Faculty of Metals Engineering and Industrial Computer Science, AGH University of Krakow, Mickiewicza 30 Av., 30-059 Krakow, Poland; ljaw@agh.edu.pl

**Keywords:** diamond, PCD, HP-HT sintering, binding phase, thermal resistance, hardness

## Abstract

A review of binder phases used for sintering diamond powders under high pressure and high temperature conditions along with an outline of the properties of polycrystalline diamonds or composite materials intended for cutting tools, wire drawing dies, and drilling rocks are presented. The interaction of diamond with metals from group VIII of the periodic table, carbon-forming metals, carbides, MAX phases and with silicides, borides, and alkali carbonates is presented. The interaction of the bonding phases with diamond was determined. The influences of sintering process parameters, amounts, and methods of introducing of these phases on the basic mechanical properties and thermal resistance of diamond materials are analyzed. The investigated material properties are compared with the properties of commercial PCD with a cobalt and the SiC binder phase.

## 1. Introduction

The first natural polycrystalline diamonds were discovered in the 19th century; these were Carbonado and Ballas, classified in the 20th century. Carbonado is a diamond with a fine crystalline structure; it is a round polycrystalline aggregate. The dimensions of individual grains do not exceed 20 μm.

The most popular way to obtain ceramic polycrystalline materials (in a broad sense) is to heat loose or pre-compacted powders to appropriately high temperatures. In such conditions, the sintering phenomenon occurs in the system, thanks to which a set of small grains in contact with each other obtains mutual direct bonds even below the temperature needed for melting. Sintering is accompanied by shrinkage of the entire system and the transition of the loose or weakly bonded powder into a solid, mechanically durable sinter. As a result of mass transfer phenomena, the free surfaces of the system are reduced, and with it the free enthalpy (surface energy) is reduced. Sintered polycrystalline diamond (PCD) compacts are widely used as cutting tools, wire drawing dies, and drilling rocks. The specificity of the diamond sintering process is the need to carry out the process under temperature and pressure conditions of its stability [1,2]. Diamond is thermodynamically stable at room temperature, at pressures above 1.6 GPa. Katzman and Libby sintered diamond compacts with a 20 vol% of cobalt binder [3]. They used a pressure of 6.2 GPa and temperature up to 1610 °C. The hardness of these compacts was about 30 GPa. The next group of PCDs was obtained by Wendorf and Rocco by cobalt infiltration from the WC-Co substrate [4]. This method is still used today. Cobalt is a catalyst for the transition of graphite into diamond and the reverse transformation and significantly reduces the diamond sintering parameters, i.e., temperature, pressure, and sintering time. Diamond sintering with the use of the so-called metal catalyst solvent (this function is performed by cobalt) is carried out in the range of 1500–2000 °C, at pressures of 5–8 GPa, for times from 30 s to 60 min [5]. High Pressure High Temperature (HP-HT) devices of various designs and different pressure and temperature ranges are used for diamond sintering. The pressure ranges obtained in individual devices are as follows:

- for the piston–cylinder apparatus up to 6 GPa, the original design of the apparatus allowed high-temperature experiments in the pressure range between 0.5 and 4.0 GPa [6,7];
- for the Belt-type high pressure apparatus, 6–10 GPa [8];
- for the Bridgman, Toroid, and the Paris–Edinburgh apparatus, 20 GPa [9];
- for the multi-anvil high pressure apparatus up to, 100 GPa [10];
- for the Kawai-type multianvile apparatus, 65 GPa [11].

Nowadays, HP SPS (High Pressure Spark Plasma Sintering) devices have appeared, which use pulsed currents instead of alternating currents. Known HP SPS devices operate at pressures of 6 GPa for belt-type apparatus [12] and up to 8.0 GPa for the HP SPS Bridgman (toroidal) apparatus [13]. Studies have shown that the use of pulse current during sintering has a beneficial effect on limiting the deformation of the crystal lattice and limiting the graphitization process of diamond [13,14]. HP-SPS processes limit diffusion and prevent the growth of grains in sintered materials [14]. Pulse current has a beneficial effect on the surface layer of particles. The sequence of electrical discharges causes the removal of impurities absorbed on the surface of sintered particles.

In 1970, Hall presented results of sintering pure diamond in the range from 1 s to several s [15]. Obtaining polycrystalline diamond without a bonding phase, by direct transformation of graphite, requires very high pressures of 12–25 GPa and temperatures exceeding 2000 °C; this was realized through a Kawai-type multianvil apparatus [16,17]. The predominant type of contact in the free powder filling is the contact of the tip of one particle with the plane of another, although contact along the planes also occurs. During pressing of the compacts, pores between the grains are partially filled with crushed diamond particles. In the case of sintering diamonds without technological additives (binder phases), isostatic conditions are not achieved in the sintered material. Then, very highly differentiated pressures can appear in the contact zone of individual grains. During high-pressure sintering, diamond grains are in a complex state of stress, as a result of which part of the diamond crystal subjected to compression can be in a state of phase stability, while part of its surface is in a state of graphite stability. It was found that in such a situation, alongside the basic carbon phases, certain amounts of metastable phases coexist, transforming one into the other [18]. The modulus of elasticity of diamond is about 1050 GPa [19,20], so in sintering conditions, the plastic deformation coefficient of diamond is not taken into account during sintering under pressure. In the case of diamond composites, research focuses on the selection of binding phases, which should provide better resistance to high temperatures and increased mechanical properties of PCDs. The introduction of an additional phase with high temperature stability should affect the sintering process and limit graphitization of diamond powder on the one hand. In addition, the introduced phase should ensure the correct pressure distribution inside the diamond compact. Incorrect pressure distribution causes the formation of tensile stresses on the surface of diamond particles located in the intergranular spaces and causes a pressure drop in these places, which in consequence leads to graphitization of the diamond surface [21]. The bonding phase has a major influence on the phenomena occurring between the processed material and the tool blade. Incorrect selection of the tool material in relation to the material to be processed may result in the cutting process:

- Excessive wear of the tool material as a result of physicochemical reactions between the materials (mainly diffusion phenomena);
- Increased cutting resistance, causing excessive heat generation, leading to damage to the processed material and energy losses resulting from increased energy consumption by the machine tool;
- Reduced surface quality of the processed material (high roughness of the processed surface).

There are basically three methods of introducing the binder phase into the diamond powder:

- Mechanical mixing of the binder phase with the diamond powder;
- Application of coatings to the powder particles;
- Infiltration from a substrate containing the binder metal or overlay infiltration using a disk prepared from the binder phase material.

The purpose of this review was to indicate the problems encountered during the preparation of polycrystals and diamond composites with different binder phases. The main emphasis was put on such issues as sintering with the participation of the liquid phase or in the solid state, the influence of unfavorable stress state on graphitization of diamond, differences in thermal expansion coefficients between diamond and binder phase, the presence of diamond “skeleton” (due to diamond–diamond bonds), precipitation of diamond from supersaturated solutions during the HP-HT sintering, the presence of residual porosity, and weak mechanical properties of the binder phase in the case of a lack of stoichiometry. The review may help in the search for the so-called perfect tool material. The advantages and disadvantages of commercial materials with cobalt and silicon binding phases are discussed. PCD studies with other metals (not forming a liquid phase in the sintering process), such as chromium, molybdenum, tantalum, and titanium (i.e., metals forming carbides), are presented. Materials sintered in the conditions of the presence of the solid phase, i.e., in the presence of various ceramic phases (carbides, silicides, MAX phases, and borides) are presented next. The next group of diamond materials presented are materials with natural rocks, carbonates, which form supersaturated solutions with diamond carbon, from which diamond precipitates. The last is a group of materials with improved electrical conductivity due to the introduction of graphene.

## 2. Nickel, Cobalt, and Iron Binding Phases

Metals from group VIII of the periodic table (so-called iron metals) are Co, Ni, Fe, Ru, Rh, Pd, Os, Ir, and Pt [22]. Ru, Rh, Pd, Os, and Pt are expensive and are, therefore, not selected as binder phase material in PCDs. Cobalt, nickel, and iron are considered as diamond binding phases. PCDs with Co, Ni, Fe are sintered in the temperature range of 1500–2000 °C and at pressures of 5–8 GPa due to the range of thermodynamic stability of diamond in these conditions and the more favorable effect of the presence of the liquid phase of the binding phase in the form of Co, Ni, or Fe. The remaining group VIII metals are characterized by higher density and higher melting temperature, which means problems with homogenization of mixtures and sintering in the solid phase, which can cause unfavorable stress distribution inside the sintered material. Studies conducted by Shul’zenko have shown that after introducing appropriate amounts of metal additives into diamond powder and subjecting the whole to sintering, the metal melts, and the liquid phase acts as a pressure-transmitting element, and an even pressure distribution occurs inside the sintered material. At the stage of grain rearrangement, the liquid phase, filling the intergranular spaces, reduces the friction forces occurring between the grains. This, in combination with external pressure, allowing a more compacted material to be obtained [23].

The wettability of the diamond by the binding phase is of decisive importance in selecting the appropriate chemical composition of the binding phase, in the case of sintering in the presence of a liquid phase. The basis for the appropriate strength of the sintered material based on diamond with the binding phase is a strong bond between the diamond and the binding phase. Rapidly cooled under high pressure, the Ni-C liquid phase undergoes a transformation into a mixture of a solid solution of carbon in nickel and metastable nickel carbide, Ni_2_C. The higher the pressure at which the system is located, the slower the cooling should be in order to preserve Ni_2_C to room temperatures. The rapid initial cooling, followed by the slow cooling at temperatures below 727 °C, leads to the decomposition of Ni_2_C, as a result of which spherical graphite particles are precipitated and distributed in a solid solution of carbon with nickel. In this case, diamond does not crystallize from the carbon formed from the decomposition of the carbide, despite the high pressures [24].

The interaction of diamond with cobalt in high-pressure sintering conditions is slower than in the case of sintering at atmospheric pressure. This phenomenon is explained, on the one hand, by the increased stability of diamond under high pressure conditions, and on the other hand, by the slower diffusion of carbon into cobalt due to the reduction in interatomic distances in the cobalt structure [18,25,26,27,28]. According to studies on sintering diamond powders with cobalt, graphite, formed as a result of the allotropic transformation of diamond, dissolves in cobalt until a saturated solution is formed. After exceeding the conditions corresponding to the equilibrium line of Co_2_C ↔ liquid + diamond, diamond precipitates from the solution. After the diamond has precipitated, liquid cobalt can further dissolve graphite. Melted cobalt has a solubility of 11.9 at.% carbon and 13 at.% at 1500 °C [29]. Co_2_C carbide is formed starting from a pressure of 4.7 GPa with a carbon content of 0.25 moles. With increasing pressure, the melting point of the solid solution increases and the concentration of carbon in the solid solution increases at the eutectic temperature [30].

In the case of the diamond–iron system, the formation of diamond from the liquid phase is possible at pressures above 6.4 GPa and temperatures above 1850 °C [30]. When increasing the pressure to 8 GPa, a stable carbide, Fe_7_C_2_, is formed. In the case of the Fe-C system, the precipitation of diamond from the saturated solution occurs at temperatures and pressures higher than in the case of the Co-C and Ni-C systems, which determines the smaller scope of use of iron as a binding phase in sintered diamond.

Materials with a binding phase selected from group VIII of the periodic table undergo degradation during tool operation, and at elevated temperatures, graphite and cracks appear [31,32]. Cobalt is a toxic metal; it is believed that inhalation contact may cause cancer and is a cause of allergies and, hence, its participation in PCD may be dangerous both at the stage of PCD production and processing of this material [33]. In addition, PCD with cobalt is a brittle material, which causes chipping of the cutting edge in some machining processes and deterioration of the quality of the machined surface.

### 2.1. PCD with a Cobalt Bonding Phase

Recrystallization is a dominant process during the diamond sintering with the cobalt bonding phase, and it only takes place for HP-HT sintering times as long as 1 h; for shorter times weaker bonds are formed [27]. The hardness of PCD with cobalt depends on the recrystallization process duration; see Table 1. For diamond mixtures sintered for shorter durations, materials are characterized with lower hardness [5]. PCDs with lower hardness cannot be used as a cutter for hard rock drillers but can be widely used for machining non-ferrous metals. There was proved that sintering conditions of 7.7 GPa and 2000 °C for the diamond mixtures with 5 vol% cobalt was necessary to obtain material without cracks [34]. However, for the sintering of diamond with the continuous cobalt film (obtained by the magnetron sputtering method), at these same conditions, 1 vol% of cobalt is sufficient to obtain a well-sintered material [34]. Lima et al. showed that the hardness of the obtained PCD sintered with a cobalt binding phase depends on the sintering time [5]. German work [35] confirmed these relationships, and it covered in more detail the kinetics of sintering diamond with the cobalt phase, including in the conditions of liquid cobalt infiltration.

**Figure 1:**
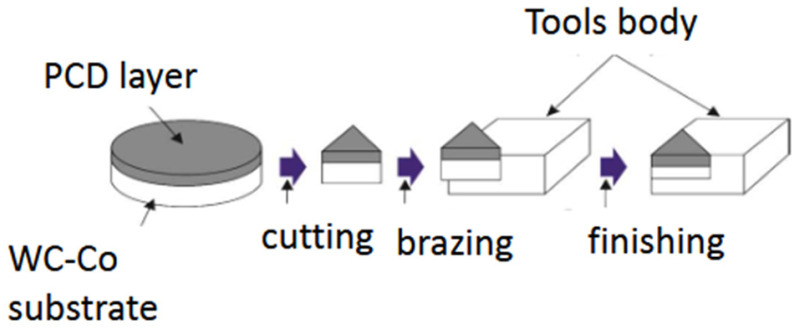
The cutting insert with a PCD blade preparation.

Cobalt is used by most large manufacturers of diamond sinters as a binding phase. It provides good mechanical properties for diamond compacts and good electrical conductivity, which allows for the use of electro-erosion methods for processing these materials. In addition, the process is carried out in relatively low pressure and temperature conditions, which gives producers tangible economic benefits. Diamond powders were sintered and bonded to the WC-Co substrate at the same time by infiltration of Co from the substrate; see Figure 1. Cobalt infiltrates the compacted diamond by capillary forces. The carbide substrate is very useful for tool producers because of the possibility of diamond brazing to the tool body.

Sufficiently high wettability of diamond materials by molten metal fillers is the principal requirement for successful brazing. The most popular commercial PCDs are two-layer materials with a cobalt phase; see Figure 1. The WC-Co substrate geometry has a significant effect on the interface of WC-Co with diamond layer and had a significant impact on the interfacial bonding strength and toughness of PDC [36,38]. The use of laser cutting allows for a complex socket in the tool body to be made and the reproducing of this shape on the PCD, and, as a result, the connection of the PCD with the tool body is strengthened [39,40]. Through the proper granulometric composition of diamond grains (e.g., differentiation of the size of diamond particles) and the selection of the introduced binding phase, an increase in the mechanical strength of diamond sinters is achieved. Traditionally, tools for the metal cutting industry are classified into four groups: superfine, fine, medium, and coarse (extra fine, fine, medium, and coarse) PCDs. It has been found that with the increase in the size of the diamond grain, the abrasion resistance increases; however, with the increase in the size of the diamond, other important properties deteriorate, such as quality and resistance to chipping of the blade and the surface quality of the processed material. Fine-grained PDC has higher abrasion resistance and coarse-grained PDC has higher toughness. The size of the diamond particles used determines the way in which the polycrystalline diamond fractures. For smaller particles of a few micrometers, fractures along grain boundaries are observed. For larger diamond particles, transcrystalline fractures are observed [41,42,43]. Hence, individual groups of tools are dedicated, for example, to finishing or roughing. Materials obtained with large diamond particles are more temperature resistant than materials with smaller diamond particles [44].

The disadvantages of PCD with a cobalt bonding phase include the tendency of catalytic graphitization of diamond and chipping and microcracking, which are the result of the thermal stresses caused by the difference between thermal expansion coefficients of diamond and the bonding metal. For diamond, at room temperature, the thermal expansion coefficient is about α = 1.0 × 10^−6^/K [45], and for cobalt it is 12 × 10^−^^6^/K [38,46], and for WC it is 3.84 × 10^−6^/K [47].

### 2.2. Thermal Stability of PCD with a Cobalt Bonding Phase

The graphitization process of synthetic diamond begins at a temperature of approximately 750 °C. The diamond oxidation process begins at lower temperatures than the graphitization process. During oxidation, gas is released in the form of carbon oxides, which adversely affects the integrity of the PCD microstructure [48,49]. Studies of PCD with a cobalt bonding phase confirmed that at a temperature of 800 °C, in the air, graphitization, oxidation, and microcrack formation occur [50]. At temperatures higher than 900 °C, the graphitization rate increase and a thin graphitized layer is formed [47]. Thermal studies of commercial PCDs with a cobalt bonding phase in the air and in a vacuum were realized [50]. In the case of PCDs at temperatures up to 800 °C, the surface of the PDC annealed in ambient air was damaged by a mixed mechanism of graphitization, oxidation, and stress-induced microcracks. At 900 °C, only the dendritic phase Co_3_O_4_ was present on the surface of the annealed PDC [50,51]. Studies of PCD materials with WC substrates indicate that the diamond layer contains not only cobalt and diamond but also WC. This is the result of both sintering diamnd on the WC substrate and preparing diamond mixtures in mills with WC-Co coating, using WC-Co balls [47,52]. It has been found that long-term mixing in the mill causes diamond cracking, and the finer the mill, the more intense the process. This phenomenon may lead to abnormal growth of small grains, which may subsequently lead to transcrystalline cracking of diamond grains [53].

As a result, during tool operation (the machining), phases such as CoO, Co_3_O_4_, and WO_2_ appear, which weaken the mechanical properties of the PCD material. The finer the PCD particles, the more of these phases are [54]. Due to the increase in volume, these oxide phases cause an increase in stresses and defects in the PCD layer, which results in higher residual stress led to the initiation of microcracks [31]. The formation of η-phase (Co,W)_6_C in the residual cobalt preceded graphite formation in the PCD samples, after exposure at 800 °C for 30 min. Thanks to the control of distribution and shape of cobalt located in the boundaries between diamond particles, the thermal stability of PDC can be improved [55]. The metal catalyst usually remains in the polycrystalline diamond matrix, but recent works have sought to exclude cobalt from the diamond layer. The removal of cobalt from the polycrystalline diamond material improves the heat resistance of PCD but reduces the fracture toughness of these materials [41,56].

In order to remove cobalt from the diamond layer with the cobalt binding phase, leaching methods (e.g., aqua regia, acids) or electrolytic methods are used [57]. The efficiency of leaching methods is very low, and, in addition, these methods are harmful to the environment. The efficiency of electrolytic cobalt removal is higher. The surface porosity of PDC for the electrolytic cobalt removal method is about 13.5% [58]. After removing cobalt, empty spaces remain in the PCD layer, which are the source of the material’s weakness. In order to fill the free spaces in the etched PCD layer, diamond coatings obtained by the hot filament chemical vapor deposition (HFCVD) method were used. Unfortunately, discontinuities of the PCD material partially remain and cobalt diffusion into this coating from deeper zones of the material where cobalt remained was observed. Cobalt dissolves carbon from the coating [59,60,61].

## 3. Diamond Composites with Silicon

Commercial solutions with a silicon binder phase are known, intended mainly for drilling purposes. After sintering, they contain up to 40 wt% of SiC as a binder phase for diamond. The possibility of creating such materials was patented in 1991 by A.E. Ringwood [62]. His patent was a milestone in the development of diamond materials highly resistant to abrasion and high temperature. The idea of using silicon was used by most companies producing diamond materials. In the late 1990s, the first commercial diamond composites appeared, with a ceramic binding phase in the form of SiC. These materials are most often obtained by the reactive sintering of diamond and silicon mixtures or infiltration of silicon into the layer of compacted diamond. SiC is formed as a result of the reaction between carbon from diamond and silicon [63,64,65]. The melting temperature of silicon is 1410 °C, so in most diamond–silicon sintering processes, silicon is present in the liquid phase. In Ekimov et al. [66], diamond with Si was sintered at 7.7 GPa and at temperatures up to 2000 °C, using a toroid-type high-pressure apparatus. In this work, the method of infiltrating a diamond preform with liquid silicon was used. The hardness of this material is 51 GPa. High hardness was explained of the material strengthening due to the blocking of dislocations at grain boundaries [66,67]. Methods of mixture of diamond with silicon powder sintering [68] and sintering diamond powders with Si coatings [69,70] were used. Moreover, Si and C atoms form very strong tetrahedral covalent bonds. The hardness of cubic β-SiC can reach up to 41 GPa under high pressure conditions [71]. The formation of SiC in the intergranular spaces is associated with an increase in volume and results in blocking of residual porosity in these spaces, which affects the presence of internal pores and increases the surface roughness, worsening the quality of the machined surface [72]. Porosity and the presence of unreacted silicon are the reasons why this material is not used in precision machining as it causes deterioration of the quality of the machined surface. Diamond materials with SiC are characterized by higher temperature resistance when compared to materials with a cobalt binding phase, having a lower brittleness and a higher compressive strength. Diamond materials with a SiC binding phase have high thermal conductivity. The SiC binding phase expansion coefficient is similar to that of diamond. The hardness of these composites is lower when compared to PCD with cobalt. These materials are very resistant to abrasion. These kind of composites have relatively low fracture toughness, <6 MPa·m^1/2^ [73]. Qian and Zhao prepared the diamond mixtures with Si using milling in a planetary mill, and they performed sintering of the mixture at 5 GPa to about 8 GPa and a temperature of about 1100 °C to about 2100 °C [74]. They obtained fracture toughness for this material of 12 MPa·m^1/2^. A. Ekimov [66] obtained diamond materials by liquid silicon infiltration of the nanocrystalline diamond preformed at 7.7 GPa and at 1400–2000 °C. The fracture toughness of this material was 10 MPa·m^1/2^, but the composite was only partially densified.

There is the possibility for the improvement of mechanical properties and resistance to high temperatures of these materials. There was sintered a mixture of diamond with 9.9 wt% Si powders with the addition of 0.1 wt% n-layer graphene (flakes of size less than 10 μm, thickness of 1.0–1.2 nm) [75]. There is no free silicon in the phase composition of the material after the sintering process. In the phase composition of these materials, there is no presence of free silicon. Graphene reduces the porosity of these composites and influences the binding of free silicon. There is an increase of 35% in the strength by, and wear resistance is seven times higher than the samples produced without the addition of graphene [75].

## 4. Diamond PCD with Other Metals

The examples in the A.E. Ringwood patent show diamond sintering with Si, Ti, Ir, W, V, Mo, and Rh binding phases [62]. There were problems with obtaining homogeneous mixtures due to the difference in metals and diamond density. Studies on obtaining diamond with silicon confirm that the basic problem in these materials is the residual porosity resulting from the blocking of some of the free spaces between the diamond particles by the formation of carbide phases (with higher volume) despite the sintering process involving the liquid phase in the form of molten silicon [73]. In the chromium–diamond system, at a pressure of 4.3 GPa, the chemical interaction begins at a temperature of 800 °C. Phase studies show the presence of Cr_23_C_6_ and Cr_7_C_3_ carbides. At a temperature of 1000 °C, the intensive formation of Cr_3_C_2_ carbide begins, which is formed by rearrangement of Cr_23_C_6_ and Cr_7_C_3_ carbides. At this temperature, the Cr_23_C_6_ carbide no longer occurs. At a temperature from 1300 °C, only the Cr_3_C_2_ carbide occurs. The interaction zone in the chromium–carbon system is much larger than in the titanium–carbon system [76]. A PCD with improved thermal stability was obtained with 90 wt% diamond and 10 wt% Mo. Samples were sintered at 7.7 GPa, using the toroidal apparatus. Experiments were performed at different temperature conditions of 1650 °C, 1750 °C, and 1850 °C. The obtained materials had thermal resistance to graphitization and oxidation, about 200 °C higher than in the case of conventional PCD. Higher thermal parameters observed for PCD-Mo may be due to the formation of in situ carbides (MoC and Mo_2_C), which inhibit the development of graphitization and oxidation processes [77,78,79]. In the case of PCD with 2.5 wt%, 5 wt%, and 10 wt% niobium content, obtained at a temperature of 1750 °C and a pressure of 7.7 GPa, an increase in the material strength and a reduction in graphitization effects were observed through the formation of NbC and NbC_0.84_ [80,81,82]. Tantalum in the amount of 2.5, 5.0, 7.5, and 10 wt% were homogenized in a planetary ball mill and sintered at 1750 °C and a pressure of 7.7 GPa. TaC formation was detected at PCD interfaces after sintering. As a result of the low chemical reactivity to the formation of Ta with W compounds, there is low tungsten contamination of the PCD [83].

### Diamond Composites with Ti Bonding Phase

Studies on the diamond–titanium system have shown that with an increase in pressure from 4.3 to 7.0 GPa for a temperature of 700 °C, the carbide formation reaction is inhibited. With an increase in pressure, the rate of carbon diffusion in titanium decreases [84]. Titanium carbide has excellent mechanical properties and has long been used for tool applications. Studies conducted by X-ray diffraction have shown that the non-stoichiometric TiC_0.85_ carbide with a defective structure is formed in the process of sintering diamond micropowders with titanium at 7.5 GPa and 1550 °C. The melting point of titanium is 1668 °C. In this case, sintering occurs in the solid phase. The filling of the space between diamond particles occurs as a result of plastic deformation of the metal [85]. The non-stoichiometric chemical composition of the carbide affects the deterioration of the mechanical properties of the PCD. By sintering diamond powder with 5 wt% titanium, materials of very high hardness (58.9 ± 2.1 GPa) and relatively high strength in a diametric compression test (141.37 MPa) were obtained. This PCD is characterized by high values of residual stresses [86]. In the case of high-pressure sintering, there is evidence to confirm the occurrence of residual stresses in diamond composites, due to the increase in volume due to the formation of carbide, the difference in the thermal expansion coefficients between diamond and TiC, as well as mechanical stresses resulting from the pressure of the press. The result of the occurrence of stresses in the sintered diamond composite is, among other things, the allotropic transformation of diamond into graphite. As a result of the uneven distribution of stresses inside the material, part of the diamond surface appears in a condition corresponding to the thermodynamic stability of graphite and graphite appears. A properly selected binding phase allows for the binding of carbon, e.g., to the form of carbide. Other forms of stress energy relaxation are microcracks and cracks that appear in the material both at the stage of its sintering and after the sintering process, most often during material processing or from the work of the blade (e.g., during turning) [86]. Studies have been carried out on the introduction of titanium into diamond and the effect of the method of metal addition on the microstructure and properties of PCDs [85]. Titanium was introduced by the mixing method in the form of 4 wt% titanium powder and 4% titanium (after decomposition) from titanium dihydride, and a Ti coating was obtained by the PVD method [85]. Materials were sintered at a pressure of 7.5 GPa, at 1550 °C, for 60 s, using the toroidal apparatus. The microstructure of the material obtained from diamond powders with Ti PVD coatings is characterized by a uniform distribution of titanium. Phase studies have shown a lower share of graphite in the material compared to PCD obtained by the method of mixing with powders. The material is characterized by the highest hardness in comparison with materials in which titanium was introduced by powder mixing. On the other hand, the predominance of less rigid diamond–TiC–diamond connections compared to diamond–diamond connections determines its lower strength and lower resistance to wear. An intensive influence of titanium from the decomposition of titanium dehydrate on the mechanical properties of the composite was found. Titanium from the decomposition of the titanium dihydride at 600 °C, in a vacuum, increases the degree of saturation of TiC formed at grain boundaries. The lattice parameter of TiC increases. The compressive strength is 19% higher than that of the material obtained from a mixture of diamond and titanium powder. PCDs, in all tested technological variants, contained internal pores. The titanium phase fills part of the intergranular spaces mainly due to plastic deformation. These spaces are also filled by diamond grains crushed by external pressure [87,88].

## 5. Solid State Sintered Diamond Composite

In the case of diamond composites, works focus on the selection of high-melting binder phases, which should provide better resistance to high temperatures and increased mechanical properties. Considering the intended use of the materials under study, the introduced phase should ensure an increase in the graphitization temperature and ensure the correct pressure distribution inside the diamond sinter. An incorrect pressure distribution causes the formation of tensile stresses on the surface of diamond particles located in intercrystalline spaces (voids) and causes a pressure drop in these places, which, as a consequence, lead to graphitization of the diamond surface. The binder phase in the diamond composite should be characterized by plasticity in the range of sintering temperatures. The plastic binder phase fills the free intercrystalline spaces and, thus, ensures a more uniform stress distribution in the material. The studies conducted for TaC and NbC composites confirmed the unfavorable effect of the lack of plasticity (resulting from the high melting temperature and high hardness of these metals) of the binder phase on the stress state of the sintered material [89]. The microstructure of these composites is characterized by the presence of a “network of cracks”. From analyzing the melting temperatures, thermal stability, and hardness of the covalent carbides (NbC (3600 °C), TaC (3800 °C), TiC (3100 °C), and SiC (2650–2950 °C)), it is obvious that the sintering process takes place in the solid phase. A complex stress state is created, which, above all, is a result of the difference in thermal expansion coefficients between diamond and carbides and the constant external load during the sintering process. The thermal expansion coefficients are as follows: for NbC it is 6.6 × 10^−6^ 1/K; for TaC it is 6.3 × 10^−6^ 1/K; for TiC it is 7.7 × 10^−6^ 1/K; and for SiC it is 4.5 × 10^−6^ 1/K [90]. The consequence of the complex stress state inside the material is their relaxation in the form of cracks [89]. Table 2 presents the hardness and Young’s modulus of diamond composites obtained at a temperature of 1800 °C and a pressure of 8.0 GPa using a toroidal apparatus. The composite was sintered from a diamond mixture (3–6 μm).

### 5.1. Diamond–SiC

The mixture of diamond (0.1–1 μm) with 10–50 wt% SiC (0.1–1 μm) and 1 wt% Al (3 μm) was sintered at a pressure 6.0 GPa and temperatures of 1400–1600 °C for 60 min in the belt-type apparatus. Through the addition of Al, the formation of graphite was limited and increases the relative density of the diamond–SiC composite. Mainly β-SiC and trace amounts of α-SiC were detected in the composite material [91]. The diamond–SiC mixture composed of microcrystalline diamond (3–6 μm) with microcrystalline 30 wt% SiC (about 5 μm) was sintered at a pressure of 8.0 GPa and a temperature of 1800 °C [92,93]. Very good cutting properties (components of the total cutting force and roughness of the surface of the processed material) were obtained. However, this carbide does not become crushed or undergo plastic deformation during the sintering process, and the complex state of stress in the material initiates the allotropic transformation of diamond into graphite. The result of the increased share of graphite in these composites is lower abrasion resistance. For these composites, at the grains, graphite strips occur as a result of the diamond graphitization process [93].

### 5.2. Diamond with TiC

Diamond powders of 3–5 μm with addition of 5 wt% of the binding phase in the form of TiC_0.92_ (3 μm) were sintered. A higher hardness, HV1, of 68.7 ± 1.8 GPa was obtained compared to the PCD with 5 wt% of Ti; see Table 3. Materials with the addition of TiC powders are characterized by a less defected structure and better mechanical properties compared to composites in which Ti or TiH_2_ powder were used [94]. The filling of the intergranular spaces in the diamond composite by the crushed titanium carbide (as a result of pressure) improves the pressure distribution in the sintered material. TiC crushing under the influence of pressure results in the decrease in the mechanical strength of the diamond–TiC composite at high temperatures. Studies of residual stresses indicate a decrease in their size, with an increase in the share of the binding phase. Reducing stresses in the material is very beneficial due to the limitation of the possibility of stress relaxation in the form of cracks [89]. The use of a larger amount of the binding phase, i.e., 30 wt% TiC, reduces the residual stresses in the surface layer of the diamond–TiC composite compared to materials with a smaller share of the binding phase. In the case of using 5% Ti or TiC in the composite (initial composition), the stress values are similar [89].

The highest hardness, HV1, of 68.7 GPa was obtained for the sinter with 5% TiC_0.92_. In the case of diamond–TiC composites with an increased share of the binding phase, for 20% TiC_0.92_, the hardness, HV1, is 35 GPa. The carbide stoichiometry changed from the initial value of TiC_0.92_ to TiC_0.95_.

Diamond (2–4 μm) with 50 wt% stoichiometric TiC or TiC_0.6_. was sintered at a pressure of 6.5 GPa and a temperature >1800 °C [95]. They obtained a homogeneous material for TiC_0.6_. The HV0.5 of these composites was about 45 GPa. After heating to 1400 °C, the material did not show any cracks or significant reduction in hardness [95]. TiC was formed via the reaction of TiC_0.6_ and carbon atoms of diamond and the crucial importance of carbon diffusion between diamond and carbide.

**Table 3:** Stoichiometry, hardness, compressive strength, and residual stresses of diamond composites with Ti and TiC binding phases [86,89].

Binding Phase	Content wt%	Stoichiometry of TiC After Sintering	Hardness HV1	Compressive Strength MPa	Residual Stresses *
TiH_2_	5	0.85	58.9 ± 2.1	-	TiC 358
					C_D_ 594
TiC_0.92_	5	0.95	68.7 ± 1.8	-	TiC 326
					C_D_ 556
TiC_0.92_	20	0.95	56.9 ± 4.6	139	-
TiC_0.92_	30	0.95	47.8 ± 2.5	108	TiC 289
					C_D_ 342
TiC_0.92_	40	0.95	35.0 ± 2.5	53	-

* The residual stress tests were carried out using the sin^2^ψ method for samples in the surface layer. The grains in the surface layer should be subjected to stresses of the same magnitude, but due to differences in the phase expansion coefficients, they affect the differences in stress values [86].

Residual stress measurements, presented in Table 3, show that increasing the share of the binding phase affects the compensation of stresses in diamond-based materials. High stress values indicate high material hardness. The diamond–TiC composite, regardless of whether it was obtained from the metallic phase or with the participation of TiC, does not guarantee the appropriate smoothness of the processed material. The reason is the uneven abrasive wear of the composite due to the chipping of titanium carbide grains from the intergrain boundaries [89]. Diamond compacts with 10 wt% TiC are very susceptible to the process of oxidation; their hardness drops absolutely after wear testing at 600 °C, and the coefficient of friction of these materials at room temperature is about two times higher than for a commercial PCD with Co [96]. The oxidation process of diamond compacts with 10 wt% TiC begins at 600 °C, and at 1000 °C material is oxidized.

### 5.3. Diamond Compact MAX Phases and with Silicides

**Figure 2:**
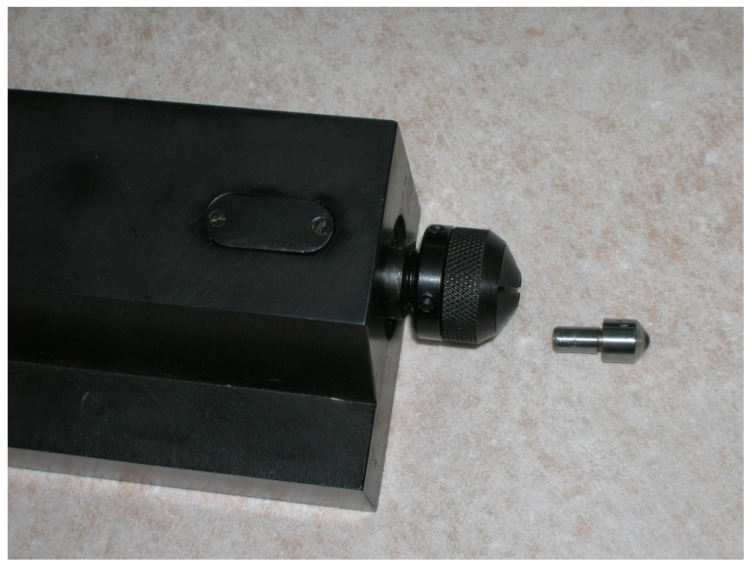
Tool for slide burnishing with spherical shape of burnishing tips obtained using from the diamond compact with 30 wt% of the Ti-Si-C binding phase.

Due to the hexagonal crystal and layered structure, Ti_3_SiC_2_ is a ceramic that exhibits significant plastic behavior. The thermal coefficient of Ti_3_SiC_2_ is about 10 × 10^−6^/K [91]. Ternary compounds (MAX phases) M_n_ + 1AX_n_, n = 1–3, (where M is a transition metal, A—carbon, boron, or nitrogen) have been studied since the 1960s. Ti_3_SiC_2_ based on MN + 1AXN “nanolaminates” might make an interesting bonding phase alternative. It is stable up to 1400–1450 °C [97,98]. Ti_3_SiC_2_ powder (2–6 μm) was used as a binding phase, assuming the possibility of filling the free intergranular spaces with this material. After sintering at a pressure of 8.0 ± 0.2 GPa and at temperature of 1800 ± 50 °C with diamond (3–6 μm), using the toroidal apparatus, the bonding phase consists mainly of Ti_x_Si_y_ with SiC and TiC crystals, with a small amount of Ti_3_SiC_2_ (because of Ti_3_SiC_2_ decomposition). The Vickers harness, HV1, for compacts with 40 wt% of Ti_3_SiC_2_ was 57.1 ± 3.00. For 30 wt% Ti_3_SiC_2_, the HV1 is 44.42 ± 3.1 GPa, and fracture toughness (measured using the notched beam method) is 8.0 MPaּ m^1/2^ [93]. The material presented good wear properties [99]. Ti-Si-C bonded composites (the Self Propagating High Temperature Synthesis (SHS) product composed of 52 wt% Ti_3_SiC_2_, 27 wt% TiSi_2_, and 21 wt% TiC) sintered at 7.8 ± 0.2 GPa and 2000 ± 50 °C were characterized by having a much higher coefficient value of friction than commercial PCDs with a Co binding phase. Similarly to the composites with 10 wt% of TiC, for the diamond composite with 10 wt% Ti_3_SiC_2_, the oxidation process begins at 600 °C, and at 1000 °C the material is oxidized. SiO_2_ starts to form above 600 °C [100,101]. There is high elongation of materials because of the presence of TiC [96]. It was proved that TiO_2_ formation contributes to material swelling and WO_3_ (W is present from the milling process) and causes a significant increase in the coefficient of friction [96]. A material with success was used for the slide diamond burnishing process; see Figure 2 [102,103,104]. Because of good electrical conductivity, the spherical shape of burnishing tips from the 30 wt% Ti-Si-C bonded composites was formed by electrical discharge machining (EDM). The materials show excellent results for this application due to their good tribological properties and high compressive radian strength of 254 MPa [92].

**Figure 3:**
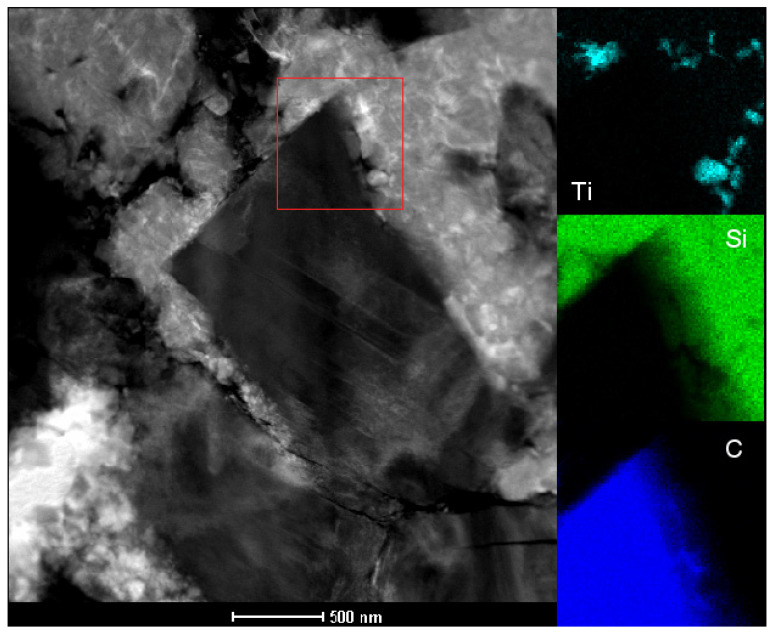
Microstructure of a diamond composite with the addition of 15% Ti_3_SiC_2_ and 5% nano TiCN (TEM—transmission microscopy; bonding phase area; distribution of Ti, Si, and C elements).

Due to the residual porosity in diamond with a 30 wt% Ti-Si-C binder phase, nanopowder of 5 wt% TiCN nano (50:50, 40 ± 5 nm) was added to the 15 wt% Ti-Si-C binder phase. This material was sintered at a pressure of 8.0 ± 0.2 GPa and a temperature of 1800 ± 50 °C using the toroidal apparatus. The material with TiCN nano does not have residual porosity at grain boundaries, which was present in the material with the Ti-Si-C binding phase. The hardness, HV1, of this material is 49.6 ± 3.6. The microstructure of 15 wt% Ti-Si-C + 5 wt% TiCN is presented in Figure 3.

The diamond compact with the addition of a 15 wt% Ti-Si-C and 5 wt% TiCN nanobonding phase is highly susceptible to oxidation because of the high content of TiC, which is about 12 wt%, after the sintering process.

**Figure 4:**
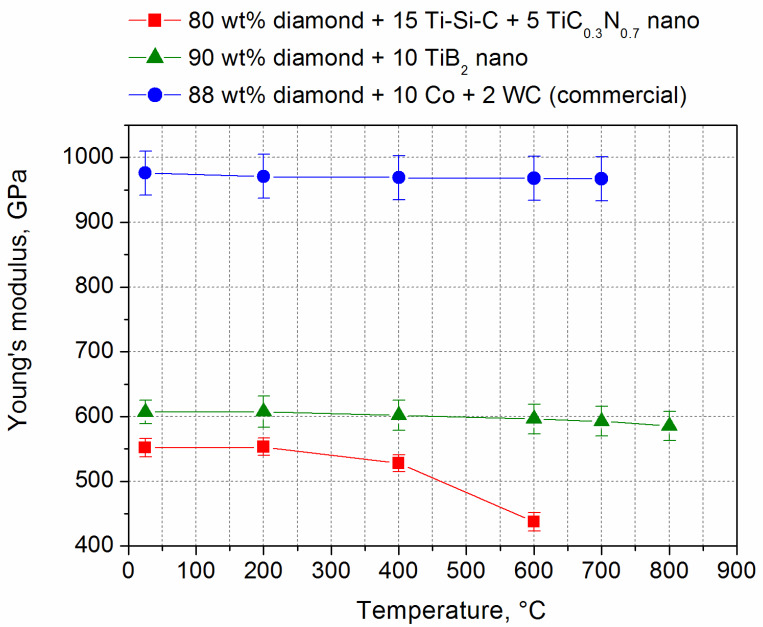
Young’s modulus during heating (air atmosphere).

The changes in Young’s modulus during heating are shown in Figure 4 [48].

The temperature in the cutting zone during machining using the diamond compact with the 15 wt% Ti-Si-C and 5 wt% TiCN nano bonding phase is substantially higher due to higher coefficient of friction. There was destruction of the tool at cutting speeds above 80 m/min. Surface roughness is not good enough for PCD-type materials [105].

Another example of the use of the MAX phase as a binding phase in a diamond composite is the Ti_3_(Si, Ge)C_2_ phase. This phase was obtained by the Self Propagating High Temperature Synthesis method. After the process, in the material there was 63.0 wt% Ti_3_Si_0.43_Ge_0.57_C_2_. The mixture of diamond powder with 30 wt% Ti_3_(Si, Ge)C_2_ was sintered with 8.0 GPa and a temperature of 1950 °C using the toroidal apparatus. The composite after sintering was composed of 2.0 wt% Ge, 4.0 wt% graphite, 4.0 wt% TiC, <1.0 wt% Ti_3_SiC_2_, and <1.0 wt% SiC. The HV1 for this material was 64 GPa [106].

For preparation of a binding phase from the Cr-Al-C system, the SHS method was used. The material was composed of 85.2 vol% Cr_2_AlC and 14.8 vol% Cr_7_C. Cr_2_AlC is a more thermally stable material than Ti_3_SiC_2_ [107]. In the material there was residual porosity, and graphite participation of 6.1 wt%. Graphite and porosity are presented at voids between diamond particles. The binding phase after sintering is composed of Cr_3_C_2_ and Al4C_3_ [108].

Molybdenum silicides (MoSi_2_ or Mo_5_Si_3_) were obtained by the self-propagating synthesis (SHS) method. The SHS binding material has a multiphase composition, composed of carbides and silicides. The melting point for MoSi_2_ is 2030 °C, and for it Mo_5_Si_3_ is 2085 °C [109]. Diamond composites containing 30 wt% of the binding phase were prepared using the toroidal apparatus at 1800 ± 50 °C and 8 ± 0.2 GPa. After sintering with MoSi_2_, the material was composed of MoSi_2_, Mo_24_Si_15_C_3_, and diamond. The hardness, HV1, for this composite was around 42 GPa. After heat treatment at 1200 °C for 30 min, in a vacuum, the HV1 decreases to 29 GPa. In the second composite containing mainly the Mo_5_Si_3_ bonding phase, the material was composed of MoSi_2_, Mo_24_Si_15_C_3_, and diamond. Although the silicides in the initial mixture are different, after sintering, both materials contain MoSi_2_, and their properties differ. No graphite was found in either sintered material. The hardness, HV1, of this material is 28.0 GPa after heat treatment, and in this same conditions it decreases to 21.0 GPa [110,111]. Diamond composites are characterized by good bonding of silicides and diamond crystallites. For Si_2_Ti, the melting point is 1470 °C. The sintering condition for 30 wt% and 40 wt% of this binding phase are different. The diamond material was sintered at 1800 ± 50 °C and 8 ± 0.2 GPa. The microstructure of the bonding phase confirms the presence of liquid during the sintering process [93]. The composite, after the sintering, was characterized by drop-shaped defected Si_2_Ti grains, long grains of SiC, prismatic crystallites of TiC, and mixtures of ultrafine grains of TiC and SiC around diamond crystallites. The hardness, HV1, of the composite with 30 wt% Si_2_Ti is 26.4 GPa, and the HV1 of the composite with 40 wt% is 50.5 GPa [112].

### 5.4. Diamond Composites with a Boride Binding Phase

Diamond powders <40 μm and a Si-18 wt%, Ti-2 wt% B alloy was sintered at 1400–1500 °C and 4.5–6.0 GPa for 50–60 s in a hexahedral pressure apparatus. The PCDs are composed of β-SiC and TiC or f-SiC and a “new phase” (which has not been clearly characterized). Yao et al. found that boron atoms in this material could improve the thermal stability of diamond [113].

TiB_2_ has superior physical and chemical properties, including a high melting point of 2950 °C; high hardness, HV0.5, of 25 GPa; good electrical conductivity (*ρ* = 20 ÷ 10 μΩcm); and excellent chemical stability [114,115]. Diamond powders (3–6 μm) and TiB_2_ powders of below 4.5 μm were sintered at 1950 ± 50 °C and 8.0 ± 0.5 GPa for 90 s using the toroidal apparatus. Different amounts in the range from 10 to 30 wt%, were used. The highest values of Vickers hardness and Young’s modulus, HV1, 46.0 GPa and 552 GPa, were obtained for diamond composites with 10 wt% TiB_2_ of the bonding phase. The phase composition analysis confirmed that this material is composed of 87.3 vol% diamond, 7.8 vol% TiB_2_, 4.7 vol% graphite, and 0.2 vol% TiC. The increase in the share of TiB_2_ in composites causes an unfavorable increase in the friction coefficient [116]. S. Cygan et al. confirmed that after sintering diamond with 10 wt%, at a temperature of 2100 ± 50 °C under a pressure of 7.8 ± 0.2 GP for 40 s, a small amount of TiB_2_ was decomposed and TiC and WB were formed [96]. In the material there was below 2 wt% of graphite and W_2_C. The presence of tungsten is the result of preparing mixtures of diamond and the binding phase in mills with hardmetal bowls and grinders. A temperature of 600 °C is critical for the diamond with 10 wt% TiB_2_ composite oxidation [96]. XRD investigations confirmed for the composite at 1000 °C that the boride and carbide phases are oxidized, but WB is present within the material. At temperatures above 1000 °C, crystalline TiO_2_ was formed along with gaseous B_2_O_3_ [117]. A similar composite was prepared using diamond powder (3–6 μm) with titanium diboride nanopowder (about 100 nm) mixtures. Nanopowders in the range of 10, 20, and 30 wt% were mixed with diamond powder at a temperature of 1950 ± 50 °C and pressure of 8 ± 0.5 GPa using the toroidal apparatus. The hardness of the material with 10 wt% nano TiB_2_ was HV1 49.7, and this was similar to the material with 10 wt% TiB_2_ micro (46 GPa). The porosity for this material is about 0.83% [116,118].

It is possible to obtain the diamond 10 wt% TiB_2_ composite from elemental powders—diamond, titanium, and boron. The sintering process was realized using two methods; the HP-HT and the HP SPS. After sintering of the diamond and Ti + 2B mixtures by the HP SPS method, a greater amount of non-defected TiB_2_ phase was formed in comparison to the compacts obtained using the HP-HT method, and in the HP SPS compacts, less graphite is present. The use of pulsed current in the HP SPS sintering results in lower level of lattice strain, which has an influence on the lower disorder of the crystalline structure of HP SPS-obtained materials [13]. The diamond composite with 10 wt% TiB_2_ is more resistant to oxidation than diamond with 10 wt% TiC or a 10 wt% binding phase from the Ti-Si-C system [90]. Because of residual porosity, the next group of composites was the sintered mixture of diamond powder (3–6 μm) with 5 wt% TiB_2_ (<100 nm) and 2 wt% Co (0.5–1.5 μm). Before sintering, the powders were treated at 600 °C and sintered at 8.0 ± 0.2 GPa and 2000 ± 50 °C using the toroidal apparatus. The material composition after sintering was composed of 88.1 wt% diamond, 7.3 wt% TiC, 0.7 wt% TiB_2_, and 3.9 wt% W_2_CoB_2_ (tungsten is present from the milling process). TiB_2_ participates in W_2_CoB_2_ formation as a donor of boron. W_2_CoB_2_ is a superhard material and is characterized by good wear resistance [118]. The composite is characterized a high hardness, HV1, of 66.6 ± 2.9 GPa due the reduction in porosity. The thermogravimetric studies showed that the decrease in the mass of the composite with TiB_2_ and Co starts at temperature 877.2 °C, which is about 70 °C higher than for the commercial PCDs (88 wt% diamond + 10 wt% Co + 2 wt% WC). The friction coefficient for the material with 10 wt% TiB_2_ is lower than for commercial material, but for 5 wt% TiB_2_ + 2 wt% Co, this is about two times higher [119].

The above-mentioned studies have influenced the attempt to eliminate tungsten as an impurity in diamond mixtures; for this purpose, ultrasonic mixing in ethylene was used. Diamond (3–6 μm) with 10 wt% of TiB_2_ (<100 nm) was sintered at a pressure of 8.0 ± 0.2 GPa and at a temperature of about 2000 °C using the toroidal high-pressure apparatus. The use of the ultrasonic mixing method eliminates the problem of oxidation of carbide phases in materials in the working environment being too rapid [52]. The influence of ZrB_2_ addition to PCDs on their properties was analyzed [120]. They observed conversion of cobalt to Co_2_B and Co_23_B_6_ compounds and the volume graphitization of these materials at 1000 °C, whereas for standard PCD it is 850 °C. The ZrB_2_–PCD tools showed excellent wear behavior [120]. An interesting solution is the use of B coatings obtained by the magnetron sputtering method, which constitute a chemical barrier on the diamond particles, to limit the interaction of diamond with cobalt during sintering with WC-Co substrates. During the sintering process on the diamond particles at 1300–1400 °C and 5–6 GPa, in a cubic high-pressure apparatus, B_4_C is formed [121]. This material has been found to have greater thermal stability than PCD with cobalt.

## 6. Alkaline Carbonate Binding Phases in Diamond Composites

Carbonates were used for diamond synthesis [122,123]. Synthesis was characterized by the greater period of diamond nucleation and smaller growth rates in comparison to transition metals such as Fe, Ni, Co, and Mn. The best catalytic properties of alkaline carbonates has Li_2_CO_3_ [124]. PCD with carbonate was sintered at 7.7 GPa and above 2150 °C for 30 min in a belt-type apparatus. To obtain this PCD, infiltration of liquid MgCO_3_ from a layer placed above the diamond was implemented. The melting point of MgCO_3_ at 8 GPa is 2000 °C [125]. As with the cobalt binder phase, the carbon in the diamond dissolves in the carbonates and precipitates as fine diamond particles from the carbon-saturated solution [126]. PCD with MgCO_3_ during the heat treatment up to 1400 °C in a vacuum does not show graphitization or cracks [127]. The PCD had a Vickers hardness, HV2, of 70 ± 5 GPa. The materials are composed of diamond and MgO, without graphite. Decomposition of MgCO_3_ was confirmed in these materials. Diamond (20 μm) with a layer of a natural MgCO_3_ (particle size of 7 μm) was sintered at 2300 °C and 8 GPa in a multi-anvil cube press. They used the infiltration method [128]. PCD was composed of diamond and MgCO_3_. The MgCO_3_ calcination occurs at temperatures ranging from 250 to 800 °C. In PCD with a MgCO_3_ binding phase, there is no calcination. Researchers suggest that the presence of MgCO_3_ in PCD at sintering temperatures above 2000 °C is due to high pressure and high densification of this material. Westraadt et al. used the infiltration technique to introduce CaCO_3_ to the diamond powder. The melting point of CaCO_3_ at high pressures of 8 GPa is about 1800 °C. The diamond content was 94.0 ± 1.5% [126]. The transition of natural CaCO_3_ under high pressure up to 9 GPa at 2000 °C. confirmed that the material after pressing and treatment was composed of the two phases of CaCO_3_, calcite and aragonite. Calcite is the low pressure phase of CaCo_3_. The presence of calcite in these samples has not been exactly explained [129].

The infiltration method for the binder addition was used for diamond–CaCO_3_ and diamond–Li_2_CO_3_ materials using the toroid-type anvil press. The diamond particle size was 15–30 μm. The content of the CaCO_3_ phase in the sintered material was 7.0 vol%, and the content of the Li_2_CO_3_ phase was 14.0 vol.%. All diamond–carbonate compacts were sintered at a high pressure of 8.0 GPa and at 2300 °C for 3 min. For PCD materials, the wear resistance of diamond–carbonate compacts were measured using a free-diamond abrasive grinding test. The wear resistance of diamond–CaCO_3_ was 33% better than that of the polycrystalline diamond with a cobalt binding phase (PDC). The wear of diamond–Li_2_CO_3_ exceeds the polycrystalline diamond with cobalt binding phase by 27% [130]. The carbonates used in most of the studies presented are natural and, therefore, environmentally friendly. In the tested materials, no calcination of the binder phase (decomposition into oxide and CO_2_) was demonstrated during heat resistance tests, but no tests of aging of this material over time have been carried out so far. A certain problem may be the accidental contamination of natural powders, which may affect the repeatability of PCD properties for this group of materials.

## 7. Electrical and Thermal Conductivity of PCD and Composite Materials

The development of ultrasonic and laser methods for machining non-conductive materials has eliminated the barriers associated with such materials. However, it is possible to change the electrical conductivity of diamond materials with non-conductive bonding phases.

Diamond materials sintered with graphene have higher electrical conductivity [131]. The problem with the nanoparticles and graphene flakes is the homogeneity of compacts. Sintering experiments under axial loading conditions confirm that the fibers and flakes are laid perpendicular to the transfer axle in the pressure apparatus. Materials were dry mixed in two stages. In the first stage, diamond powders (24–40 μm) were mixed with diamond powder (about 300 nm) and then in the second stage mixed with graphene, Gn(4) (3–10 nm thick), and sintered at a pressure of 7.0 GPa for about 60 s and at a temperature of 1700 °C in a toroidal apparatus. The Vickers hardness, HV0.5, of this composite was 47–50 GPa, and the specific electrical resistivity was 0.76 × 10^10^ Ω cm. The hardness of composites sintered without graphene is 55 GPa, with a resistivity of 3.7 × 10^10^ Ω cm [132,133].

Properties of the PDC with a 4–10 wt% Co binding phase with PDC with Co and 0.05–0.3 wt% graphene addition were compared [134]. Materials were prepared from mixtures of diamond powders: 70 wt% of 30–50 μm, 15 wt% of 4–8 μm, and 15 wt% 1–2 μm. Graphene was mixed with polyvinyl pyrrolidone (PVP) by the ultrasonic mixing in ethanol and next with diamond and cobalt in the mill. Mixtures were annealed at 1000 °C in a vaccum and sintered at 5.5–6 GPa and 1500–1600 °C, using a large-volume cubic press. For the material, the hardness and wear resistance of the PCD with 0.1 wt% graphene were improved by 75% and 33%, respectively. The excellent electrical and thermal conductivity and mechanical properties of graphene effectively improved the comprehensive performance of the PDC. The specific electrical resistivity of the PCD with 0.1 wt% graphene was 10.04 ± 0.6 10^10^ Ω cm [134].

## 8. Summary

The basic problem occurring in PCD with a cobalt binder phase is their relatively low temperature resistance. It is possible to increase the temperature resistance by using a binder phase other than cobalt. The key properties of diamond in relation to the binding phase include the wettability of diamond by this phase (if it is a liquid during sintering), the ability to form strong chemical bonds, or the ability of diamond to precipitate from solutions supersaturated with the binding phase and carbon creating a “diamond skeleton” at the boundary with the diamond particles. It is advantageous to use plastic or liquid phases in sintering conditions, which under the influence of pressure during sintering fill the voids between diamond particles, providing an appropriate stress distribution, and thanks to these thermodynamic conditions that are favorable to diamond stability, limiting the transformation into graphite. The use of phases with higher melting temperatures requires the use of higher temperatures and pressures during the sintering process. Increasing these parameters shortens the sintering time up to 30 s. Extending the sintering time results in graphitization of the diamond. For higher sintering pressures, it is necessary to optimize the process in terms of sintering temperature and time, assuming the criteria of minimizing the graphite content, high hardness, and high Young’s modulus of these materials. Sintering diamonds with carbonates is similar from a chemical point of view to the process of sintering diamond with cobalt because the temperatures of sintering are above 2000 °C [126]. Furthermore, it takes place with the participation of the liquid phase. Carbonates dissolve the diamond carbon, creating a supersaturated solution from which diamond particles precipitate. Research shows that these materials have very good wear resistance. The use of carbide-forming metals often takes place in the solid phase, which generates stresses, and the materials tend to form graphite already in the sintering phase of the material [86]. Carbides are formed under high pressure conditions already below the melting temperature; the effect is an increase in the volume of material in the voids and the failure to fill the voids due to their partial blocking. This phenomenon is responsible for the residual porosity of these diamond composites [81]. Porosity results in greater roughness of machined materials; an example is the cutting tool of diamond with the Si binding phase. In the case of titanium or TiC as a binding phase, there is a problem of TiC cracking, which also affects the quality of the processed material. For these materials, at a temperature of about 600 °C, Ti oxidation occurs, rutile TiO_2_ appears, and the volume of the material increases significantly, which causes the formation of cracks [90]. Despite the plastic behavior of the MAX phases, the materials are characterized by the presence of graphite after the sintering process. Ti_3_SiC_2_ oxidizes at 600 °C and shows a large increase in the friction coefficient with increasing temperature. An interesting group of materials are silicides, for example, Si_2_Ti [111]. In this case, sintering takes place with the participation of the liquid phase, as evidenced by the composite microstructure. The synthesis of binding phases (for example the SHS method) before their addition to the diamond mixtures is more advantageous than the introduction of elementary powders, which react during the sintering process, often forming non-stoichiometric phases with worse mechanical properties. Diamond materials with boride bonding phases are characterized by high temperature resistance [111]. Borides have great potential as diamond-binding phases.

The PCD and diamond composites should be characterized by an even distribution of the binding phase but a sufficiently large share of diamond–diamond connections (the presence of a diamond skeleton). The research should be supported by a thermodynamic analysis of the resulting compounds, their assessment with a view to eliminating hygroscopic compounds, those that decompose at low temperatures, those that oxidize below 700 °C, etc. When conducting tests of sintered diamond materials, attention should be paid to changes in the volume and mass of materials depending on temperature (DTA/TG), and the friction coefficient depending on temperature. Very often, tests of temperature resistance of these materials are limited to tests of hardness changes after heating at a specific temperature. Hardness changes can sometimes be misleading, due to the high hardness of some oxygen or tungsten phases. Tungsten is present mostly as a contaminant in the process of preparing diamond mixtures in mills with bowls and grinding media made of WC-Co sintered carbides. The residual porosity of materials could be decreased by nanoparticle additives, and it is possible to improve the mechanical properties of diamond composites. The addition of graphene has a beneficial effect on the change in electrical conductivity and mechanical properties of diamond materials. The presence of graphene may result in better densification of diamond composites. Additives in the form of nanoparticles and graphene require special preparation of mixtures in order to obtain a homogeneous microstructure of materials, without clusters of these additives, which tend to agglomerate. As a result of the development of high-pressure devices, obtaining diamond composites with the participation of high-melting phases, at pressures higher than 6 GPa and at temperatures above 1800 °C, is possible because of possibilities of new apparatus. All the more so because the economic calculation of obtaining these materials may be comparable to PCD with cobalt, due to the shortening of the sintering time from several dozen minutes to 10 min or s. The sintering process is influenced by the volume of the sintered material, which is related to the temperature distribution inside the assembly for High Pressure-High Temperature sintering (the graphite heater).

## 9. Conclusions

The presented studies indicate that it is possible to combine several concepts and create an ‘ideal’ bonding phase. It should be a multicomponent phase, ensuring the following conditions are met: the voids between diamond particles are met; it is resistant to oxidation to, at least, a temperature of 1000 °C; it should not crumble during tool operation; it is characterized by the absence of graphite after the sintering process; it is characterized by abrasion resistance; and it has good heat dissipation during tool operation.

## Figures and Tables

**Table 1:** Content of cobalt in PCD, sintering conditions, and hardness for selected PCDs.

Diamond and the Sintering Conditions	Hardness *	Reference
Natural diamond (111)	HV 110 GPa	[19]
Diamond 6 wt% Co 7 GPa/2000 °C/1 h	HV 80 GPa	[35]
Diamond + 10% Co + 2%WC	HV 77 GPa	Commercially available material
Diamond 7 wt% Co + 1.5 wt% W 7.7 GPa/1500 °C/15 min	HV 61.60 GPa	[36]
Diamond 5 wt% Co 7 GPa/1500 °C/30 s	HV 42 GPa	[5]

* It should be realized that the accuracy of the measurement is generally unknown (measurement errors are as high as 20%) [37]. The loads at which the measurements were taken in most cases are not known.

**Table 2:** Hardness and Young’s Modulus for diamond composites with 30 wt% carbide binding phases [86] and 30 wt% carbide powders (2–3 μm) using a toroidal apparatus.

Binding Phase	Hardness HV1 GPa	Young’s Modulus GPa
30 wt% NbC	26.1 ± 2.1	323.0
30 wt% TaC	34.5 ± 1.4	289.0
30 wt% TiC	47.8 ± 2.4	504.5
30 wt% SiC	32.6 ± 4.3	366.5

## Data Availability

No new data were created or analyzed in this study.

## Abbreviations

The following abbreviations are used in this manuscript:

PCD	polycrystalline diamond compacts
HP-HT	High Pressure-High Temperature
HP SPS	High Pressure Spark Plasma Sintering
SHS	Self-Propagating High Temperature Synthesis
PVD	physical vapor deposition
